# Youth‐friendly services and a mobile phone application to promote adherence to pre‐exposure prophylaxis among adolescent men who have sex with men and transgender women at‐risk for HIV in Thailand: a randomized control trial

**DOI:** 10.1002/jia2.25564

**Published:** 2020-08-31

**Authors:** Wipaporn Natalie Songtaweesin, Surinda Kawichai, Nittaya Phanuphak, Tim R Cressey, Prissana Wongharn, Chutima Saisaengjan, Tanat Chinbunchorn, Surang Janyam, Danai Linjongrat, Thanyawee Puthanakit, the CE‐PID – TRC Adolescent Study Team, Praphan Phanuphak, Chitsanu Pancharoen, Somsong Teeratakulpisarn, Reshmie Ramautarsing, Rena Janamnuaysook, Kritima Samitpol, Jiratchaya Kongkapan, Artsanee Chancham, Pravit Mingkwanrungruang, Narukjaporn Thammajaruk, Nuttawut Teachatanawat, Krittaporn Termvanich, Tippawan Pankam, Thantip Sungsing, Sorawit Amatavete, Chotika Prabjantuek, Phubet Panpet, Jureeporn Jantarapakde, Tuangtip Theerawit, Watsamon Jantarabenjakul, Suvaporn Anugulruengkitt, Nattapong Jitrunruengnit, Noppadol Wacharachaisurapol, Pintip Suchartlikitwong, Pakpoom Janewongwirot, Juthamanee Moonwong, Rachaneekorn Nadsasarn, Patchareeyawan Srimuan, Siwanart Thammasala, Sasiwimol Ubolyam, Patcharin Eamyoung, Jiratchaya Sophonphan, Stephen Kerr, Taninee Petwijit, Chansuda Bongsebandhu‐phubakdi, Buajoom Raksakul, Pathranis Meekrua, Orawan Fungfoosri, Prapaipan Plodgratoke, Pakitta Kidsumret, Kamolchanok Chumyen, Sahakun Chintanakarn, Sirinthon Yenjit, Chanin Suksom, Tanunjit Rugphan, Somsri Tantipaibulvut, Prakaipech Kaw‐In, Penprapa Chanachon, Chanjiraporn Pondet

**Affiliations:** ^1^ Center of Excellence for Pediatric Infectious Diseases and Vaccines Faculty of Medicine Chulalongkorn University Bangkok Thailand; ^2^ Institute of HIV Research and Innovation Bangkok Thailand; ^3^ PHPT/IRD UMI 174 Faculty of Associated Medical Sciences Chiang Mai University Chiang Mai Thailand; ^4^ Department of Immunology & Infectious Diseases Harvard T.H. Chan School of Public Health Boston MA USA; ^5^ Department of Molecular & Clinical Pharmacology University of Liverpool Liverpool United Kingdom; ^6^ The Service Workers IN Group Foundation Bangkok Thailand; ^7^ Rainbow Sky Association Bangkok Thailand; ^8^ Department of Pediatrics Faculty of Medicine Chulalongkorn University Bangkok Thailand

**Keywords:** adolescents, HIV prevention, pre‐exposure prophylaxis (PrEP) adherence, men who have sex with men (MSM), transgender women (TGW)

## Abstract

**Introduction:**

Strategies are needed to curb the increasing HIV incidence in young men who have sex with men (YMSM) and transgender women (YTGW) worldwide. We assessed the impact of youth‐friendly services (YFS) and a mobile phone application (app) on adherence to pre‐exposure prophylaxis (PrEP) in YMSM and YTGW in Thailand.

**Methods:**

A randomized control trial was conducted in YMSM and YTGW aged 15 to 19 years. Participants were provided daily oral tenofovir disoproxil fumerate/emtricitabine (TDF/FTC), condoms and randomized to receive either YFS or YFS plus a PrEP app (YFS + APP), whose features included self‐assessment of HIV acquisition risk, point rewards and reminders for PrEP and clinic appointments. Clinic visits occurred at zero, one, three and six months and telephone contact at two, four and five months. HIV testing was performed at every clinic visit. PrEP adherence was evaluated with intracellular tenofovir diphosphate (TFV‐DP) concentrations in dried blood spot (DBS) samples at months 3 and 6. The primary endpoint assessed was “PrEP adherence” defined as TFV‐DP DBS concentrations ≥700 fmol/punch (equivalent to ≥4 doses of TDF/week).

**Results:**

Between March 2018 and June 2019, 489 adolescents were screened at three centres in Bangkok. Twenty‐seven (6%) adolescents tested positive for HIV and 200 (41%) adolescents participated in the study. Of these, 147 were YMSM (74%) and 53 YTGW (26%). At baseline, median age was 18 years (IQR 17 to 19), 66% reported inconsistent condom use in the past month. Sexually transmitted infection prevalence was 23%. Retention at six months was 73%. In the YFS + APP arm, median app use duration was three months (IQR 1 to 5). PrEP adherence at month 3 was 51% in YFS and 54% in YFS + APP (*p*‐value 0.64) and at month 6 was 44% in YFS and 49% in YFS + APP (*p*‐value 0.54). No HIV seroconversions occurred during 75 person years of follow‐up.

**Conclusions:**

Youth‐friendly PrEP services enabled good adherence among half of adolescent PrEP users. However, the mobile phone application tested did not provide additional PrEP adherence benefit in this randomized trial. Adolescent risk behaviours are dynamic and require adaptive programmes that focus on “prevention‐effective adherence.”

## 1 INTRODUCTION

Adolescents and young men who have sex with men (YMSM) and young transgender women (YTGW) bear disproportionate burdens of HIV globally [1, 2, 3, 4]. In Thailand, the HIV incidence among YMSM/YTGW has been consistently high, reported at 4 to 11 per 100 person years [3, 4, 5, 6, 7], similar to the United States where an estimated 14% of TGW are living with HIV (US CDC 2019) and over a third of all new HIV infections are in YMSM [8, 9]. The Thai epidemic among YMSM and YTGW is perpetuated by low rates of HIV test uptake, poor HIV knowledge and rates of condom use below 50% [10, 11]. To achieve the ending of AIDS, comprehensive HIV prevention packages must be delivered to this key population [12]. Pre‐exposure prophylaxis (PrEP) awareness in Thailand is low, reflected in national figures from December 2018, where only an estimated 4% of PrEP‐eligible YMSM and YTGW in Thailand received it, with one‐quarter and one‐third, respectively, accessing PrEP in Bangkok [13, 14]. Only 2% of all PrEP prescribed in 2017 were to under 20s despite this age group having the highest national HIV incidence of 10 per 100 person years [13].

YMSM and YTGW at risk of HIV face a multitude of barriers in accessing and adhering to PrEP, including financial, third party consent laws, low perceived HIV risk, gender‐based stigma and discrimination and lack of services that address their psychosocial needs [15, 16, 17, 18, 19]. Research studies from the United States emphasize the importance of regular contact to ensure good PrEP adherence in adolescents, with adherence dropping to less than half of PrEP users after monthly visits are extended to quarterly visits [20, 21].

Thai YMSM and YTGW face considerable issues with low PrEP uptake and adherence. Among the 2,986 Thai adolescents and young adults aged 15 to 24 years who received counselling about PrEP between 2016 and 2020, only 24% accepted PrEP [22]. Among the 714 who have cumulatively initiated PrEP nationwide, only 22% continue to take PrEP since 2016 [22]. These uptake and retention challenges are similar to those seen with previous adolescent trials network (ATN) studies in adolescents aged 15 to 19 years, with PrEP discontinuation during ongoing HIV acquisition risk more likely (0.82 per 10‐year increase in age) in younger individuals [23, 24].

Mobile health (mHealth) technologies can facilitate task‐shifting by utilizing its automation, widen availability, non‐reliance on infrastructure development and ability to deliver personalized care [25]. As the information‐motivation‐behavioural (IMB) skills model has been validated and used previously for various HIV reduction interventions, it was utilized in this study to support engagement of adolescents in increasing self‐risk awareness and consequently motivate behavioural risk reduction through *tailored information:* feedback of a calculated self‐HIV risk level based on information of weekly risk behaviours, and *motivation:* point accumulation for mobile application use [26, 27, 28]. The goal of this study was to investigate whether use of a mobile phone application (app) in conjunction with youth‐friendly services would improve PrEP adherence in YMSM and YTGW in Thailand.

## 2 METHODS

### 2.1 Study design and participants

This study was a prospective randomized controlled trial among young adolescents at risk of HIV acquisition in Bangkok, Thailand. Enrolment criteria included: (1) being YMSM or YTGW (assigned male sex at birth, self‐reported sex with men, self‐defined gender identity of being MSM or TGW for any period of time); (2) 15 to 19 years old; (3) self‐reported risk behaviours, including inconsistent condom use; (4) being HIV negative. Participants could be new, previous or ongoing PrEP users, and were screened and recruited into the study at voluntary HIV testing centres at the Thai Red Cross AIDS Research Centre (TRCARC) and its community‐based organization (CBO) branches, Rainbow Sky Association Thailand (RSAT) and Service Workers in Group Foundation (SWING), all located in Bangkok, Thailand via counsellors, online advertising, peer recruiters and word of mouth. Institutional Review Board approval was granted by the Faculty of Medicine, Chulalongkorn University with a waiver for parental consent granted. This study was registered with ClinicalTrials.gov Identifier: NCT03778892.

### 2.2 Study procedures

Participants were randomized (1:1) to receive youth friendly services (YFS) or YFS plus use of a PrEP adherence supporting mobile phone app (YFS + APP). Clinic visits occurred at months 0, 1, 3, 6 and telephone contact was made at months 2, 4 and 5. All participants were provided PrEP counselling, condoms and lubricants and daily oral tenofovir disoproxil fumarate (TDF) 300mg and emtricitabine (FTC) 200mg fixed‐dose combination tablets; TenoEm (Thai Government Pharmaceutical Organization), Tenof‐Em (Hetero Healthcare) or Ricovir‐EM (Mylan). Sexually transmitted infection (STI) screening (*Neisseria gonorrhea*, *Chlamydia trachomatis*) was performed on urine and anal swab samples and syphilis on blood samples at baseline and month 6 [29]. Surveys on sexual risk behaviours and perception were administered at baseline and monthly thereafter.

### 2.3 Youth‐friendly services only arm

YFS provided included ongoing counselling or support provided outside scheduled visits through online instant messaging or telephone calls with responses provided within 24 hours. Trained counsellors provided motivational interviewing focusing on risk reduction and adolescent self‐empowerment through collaborative, non‐judgemental discussions and positive reinforcement throughout the study [30, 31]. Visits focused on building a fun and friendly atmosphere to build rapport with participants. Clinics were available out‐of‐office hours. Topics covered in counselling sessions were tailored to individual needs and included educational issues, lesbian, gay, bisexual, transgender (LGBT) stigma and discrimination, mental health and substance abuse issues, with specialist referrals where necessary. All clients had access to transgender counselling and gender‐affirming related blood testing, and hormonal therapy.

### 2.4 Youth‐friendly services plus mobile phone application arm

In addition to YFS, the YFS + APP arm received an app for use, named “Project Raincoat” produced by Focal Intelligence Co., Ltd., which was designed based on use of the IMB skills model, specifically with information tailored to promote PrEP adherence and reduction of risk behaviours [26, 28]. We conducted two adolescent YTGW and YMSM focus group discussions (FGDs) to inform design of the app. Focus groups of participants aged 15 to 19 years were conducted using a semi‐structured interview guide with topics on desirable app functions, aesthetic designs and potential barriers and motivators for use. Key themes and subthemes were identified from a content analysis and utilized in informing app design, which included features supporting self‐risk evaluation where users could input data once weekly on number of sex acts, sex partners, pills taken and condom use which was then used to calculate a feedback HIV risk of low, medium, high and very high risk. Points were rewarded in real time for data input (maximum reward of 21 points per week) as well as responding to staff follow‐up calls (5 points each), attendance of clinic visits (10 points each) and negative anti‐HIV test results (50 points each). Points were exchangeable for cash, 100 points being exchangeable for 100 Thai Baht (3 USD). Users were able to customize self‐reminders for medication and appointment reminders. Due to budgetary restraints the app was available on the Android operating system (OS) only and those using other OSs were loaned Android OS mobile phones.

### 2.5 Data collection process

Behavioural surveys were completed by participants on paper forms at clinic visits. During scheduled telephone follow‐up sessions, these were administered by staff. Participants completed monthly surveys asking about numbers of sexual partners, numbers of sex acts, condom use, numbers of PrEP pills taken each week and self‐perceived HIV risk level. Self‐perceived risk was determined by asking participants “in the last month, how would you rate your own risk of getting HIV?”. Participants were able to select 1 from the 4 following possible responses; “low risk,” “moderate risk,” “high risk” and “extremely high risk.” PrEP adherence was evaluated using tenofovir‐diphosphate (TFV‐DP) concentrations in dried blood spot (DBS) samples at months 3 and 6; with ≥700 fmol/punch equivalent to ≥4 TDF doses/week. App paradata including first and last entry into the app were collected [32].

### 2.6 Laboratory assays

HIV antibody testing was performed using a chemiluminescent immunoassay (Architect HIV Ag/Ab Combo Reagent, Abbott Laboratories, Wiesbaden, Germany or Cobas HIV Combi Principle CMIA, 4^th^ Generation). HIV testing at CBOs was performed using a rapid strip test (Alere Determine HIV‐1/2, Alere International Limited, Ballybrit Galway, Ireland). STI screening was performed on urine and anal swab specimens for *Neisseria gonorrhea* (NG) and *Chlamydia trachomatis* (CT) with nucleic acid amplification (NAAT) testing using *in vitro* polymerase chain reaction (PCR) assays (Abbott RealTime CT/NG, Abbott Molecular, Inc., IL, USA). Syphilis testing at the TRCAC was performed using the electrochemiluminescence immunoassay analyser (ECLIA) and chemiluminescent magnetic microparticle immunoassay (CMIA). Syphilis testing at CBOs was performed using a solid phase immunochromatographic assay for the qualitative detection of antibodies of all isotypes (IgG, IgM, IgA) against *Treponema pallidum* (SD BIOLINE Syphilis 3.0, Standard Diagnostics, Inc., Kyunggi‐do, Korea). TFV‐DP concentrations were measured from dried blood spot (DBS) samples collected onto Whatman Protein Saver 903 cards. Dried blood spots were stored at −70°C until analysis. TFV‐DP was analysed using liquid chromatography mass spectrometry (LC‐MS/MS). The TFV‐DP calibration curve range was 200 to 10,000 fmol/3mm punch [33, 34].

### 2.7 Sample size estimation

Estimating from results of previous adolescent PrEP trials, presuming that 45% of those in the YFS arm and 65% of those in the YFS + APP would have TFV‐DP levels ≥700 fmol/punch with a power of test of >80% and presumed 20% loss of cases to follow‐up, a sample size of 100 for each arm was calculated, equating to a total of 200 participants needed for the trial [21, 35].

### 2.8 Statistical analysis

Primary outcome was PrEP adherence as measured via TFV‐DP at months 3 and 6 follow‐up. PrEP adherence was defined as those who had TFV‐DP levels of ≥700 fmol/punch [36]. Secondary outcomes included rates of HIV infection, rates of study retention at month 6, associated factors with PrEP adherence and overall HIV acquisition risk protection (combined period with PrEP adherence or 100% consistent condom use).

Consistent condom use was defined as 100% condom use during all episodes of sexual intercourse. Substance use was defined as substances taken recreationally, including alcohol, sildenafil citrate, poppers and other illicit drugs (including amphetamine, ketamine and marijuana). Loss to follow‐up was defined as no attendance for clinic visits or response to scheduled telephone calls for at least two consecutive months.

Associations of PrEP adherence with factors at enrolment was examined using univariable and multivariable logistic regression models. Associations were presented using odds ratios and 95% CI with *p*‐values calculated utilizing the Z‐test (Wald test). Factors where an association of *p* < 0.1 in univariable analysis were selected for further multivariable analysis.

Age analysis was divided into 15 to 17 to represent “adolescence” and 18 to 19 to represent “young adulthood” [37]. To assess level of HIV protection from either PrEP adherence and/or 100% condom use, behavioural risk data were summarized into three‐month blocks of risk periods prior to TFV‐DP collection. Stata/SE 13.0 was used for all data analyses.

## 3 RESULTS

### 3.1 Characteristics at baseline and month 6

Between March 2018 and June 2019, 489 adolescents were screened, 27 (6%) tested positive for HIV and 200 (41%) enrolled to the study. There were 147 were YMSM (74%) and 53 YTGW (26%). Baseline characteristics between the YFS and YFS + APP arms were similar (Table 1). Median (IQR) age was 18 years (17‐19) at enrolment. Median (IQR) age of sexual debut was 16 (15‐17) years. Of those sexually active within the past month, 34% reported consistent condom use. Laboratory diagnosed STI prevalence at enrolment was 23%. Thirteen percent of participants overall reported substance use in the last three months. Self‐reported substance use included alcohol (6%), amphetamines or methamphetamines (4%), ketamine (0.5%), poppers (volatile alkyl nitrites) (5%), marijuana (2%) and sildenafil citrate (4%).

**Table 1:** Baseline characteristics of adolescents taking once daily HIV pre‐exposure prophylaxis

	Overall, n = 200 (%)	YFS, n = 100	YFS + APP, n = 100
Gender identity			
MSM	147 (74%)	71 (71%)	76 (76%)
TGW	53 (26%)	29 (29%)	24 (24%)
Age at enrolment median (IQR)	18 (17, 19)	18 (17, 19)	18 (17,19)
15 to 17 years	63 (32%)	32 (32%)	31 (31%)
18 to 19 years	137 (68%)	68 (68%)	69 (69%)
Age sexual debut (years)	16 (15, 17)	16 (15, 17)	16 (15, 17)
Sexually transmitted infections^a^	45 (23%)	19 (19%)	26 (26%)
No. of sex partners in the past month			
0	57 (29%)	29 (29%)	28 (28%)
1	87 (44%)	41 (41%)	46 (46%)
≥2	56 (28%)	30 (30%)	26 (26%)
Condom use in the past month in sexually active (N = 143)			
Inconsistent	94 (66%)	46 (65%)	48 (67%)
Consistent	49 (34%)	25 (35%)	24 (33%)
Substance use in the past 3 months^b^	26 (13%)	8* (8%)	18* (18%)
Self‐perceived risk (N = 197)			
Low risk	121 (61%)	62 (63%)	59 (60%)
Moderate risk	66 (34%)	29 (30%)	37 (37%)
High‐ extremely high risk	10 (5%)	7 (7%)	3 (3%)

Consistent condom use = 100% condom use. MSM, men who have sex with men; TGW, transgender women; YFS, youth‐friendly services; YFS + APP, youth‐friendly services plus pre‐exposure prophylaxis adherence motivation mobile phone application use.

^a^ Sexually transmitted infections were laboratory confirmed and included syphilis, *Neisseria gonorrhoea* and *Chlamydia trachomatis*, the latter 2 from anal swabs and urine samples

^b^ Substance use included alcohol, amphetamines, methamphetamines, ketamine, poppers (volatile alkyl nitrates), marijuana and sildenafil citrate

* *p *< 0.05 with Pearson’s chi‐squared test.

An overall six‐month retention rate of 73% was observed at six‐month follow‐up, 72% in the YFS arm and 73% in the YFS + APP arm (*p* = 0.87). No characteristics were found to be associated with month 6 retention (Table 2). Consistent condom use in the past month among sexually active adolescents increased from 34% (95% CI, 25 to 43) at baseline to 58% (95% CI, 49 to 68) at month 3 (*p* < 0.001) and remained a similar level at month 6 (52%; 95% CI, 41 to 62). No evidence of reduced condom use was seen with good PrEP adherence (Table 3). STI incidence was 25.2 per 100 patient years during the study. In the 75 person‐years of follow‐up, there were no incidence cases of HIV seroconversion.

**Table 2:** Characteristics associated with retention^a^ at 6 months

Characteristics	No	Retention (%)	Unadjusted odds ratio (95% CI)	Wald test *p*‐value
Gender identity				
MSM	147	109 (74.2)	1.35 (0.68, 2.69)	0.39
TGW	53	36 (67.9)	1	
Age at enrolment (years)				
15 to 17	63	44 (69.8)	1	
18 to 19	137	101 (73.7)	1.21 (0.63, 2.34)	0.57
Education				
≤Junior high school	87	65 (74.7)	1	
≥Senior high school	113	80 (70.8)	0.82 (0.44, 1.54)	0.54
Number of sex partners in last month				
0	57	43 (75.4)	1	
1	87	63 (72.4)	0.85 (0.04, 1.84)	0.69
≥2	56	39 (69.6)	0.75 (0.33, 1.71)	0.49
Condom use in last month among sexually active (N = 143)				
100% condom use	49	34 (69.4)	1	
Inconsistent condom use	94	68 (72.3)	1.15 (0.54, 2.46)	0.71
Substance use in last three months^b^				
No	174	122 (70.1)	1	0.06
Yes	26	23 (88.5)	3.27 (0.94, 11.36)	
Self‐perceived risk (N = 197)				
Felt at low risk	121	92 (76.0)	1	
Felt at moderate‐extremely high risk	76	53 (69.7)	0.72 (0.38, 1.38)	0.33
Intervention arm				
YFS	100	72 (72.0)	1	
YFS + APP	100	73 (73.0)	1.05 (0.56, 1.96)	0.87

MSM, men who have sex with men; TGW, transgender women; YFS, youth‐friendly services; YFS + APP, youth‐friendly services and mobile phone application.

^a^ Retention defined as engagement with PrEP prevention services throughout six‐month study period with no more than 1 period of disengagement for no longer than 2 consecutive months. PrEP, pre‐exposure prophylaxis

^b^ Substance use defined as alcohol, amphetamines, methamphetamines, ketamine, poppers (volatile alkyl nitrates), marijuana and sildenafil citrate.

**Table 3:** Proportions of adolescents with 100% condom use over time stratified by PrEP adherence at months 3 and 6

	Baseline^a^	Month 3 visit	*p*‐value	Baseline^a^	Month 6 visit	*p*‐value
Overall^b^, ^c^	38 (34%, 25 to 43)	66 (58%, 49 to 68)	0.0002	33 (33%, 24 to 42)	48 (52%, 41 to 62)	0.008
PrEP adherent^d^	21/67 (31%, 20 to 42)	36/60 (60%, 48 to 72)		17/50 (34%, 21 to 47)	27/49 (55% 41 to 69)	
Non‐PrEP adherent	17/45 (38%, 24 to 52)	30/53 (57%, 43 to 70)		16/50 (32% 19 to 45)	21/44 (48%, 33 to 62)	

^a^ Baseline figures based on availability of TFV‐DP results to match with self‐reported condom use data for corresponding follow‐up month analysed

^b^ *p*‐value of Z‐test for proportions comparing % of consistent condom use between month 3 versus month 6 = 0.329

^c^ No *p*‐values calculated from Z‐test for proportions comparing % of consistent condom use between PrEP adherent versus non‐adherent groups within visits reached 0.05

^d^ PrEP adherent defined as tenofovir‐diphosphate levels ≥700 fmol/punch.

### 3.2 Mobile application use

Of the 100 participants assigned to use the app, 62% had their own phone and the remaining 38% were loaned phones. Eighty‐seven percent of participants used the app more than once and median duration of app use was three months (IQR 1 to 5). Approximately 75% of participants completed self‐risk assessments in the first two weeks, dropping to 50% in six weeks and 25% by week 12. For point rewards, 60% of adolescents earned 100 to 199 points in the app, with a median of 120 points earned (IQR 60 to 205). One third (32%) redeemed their cash rewards at their month 3 visit and the remaining at their month 6 closing visit.

### 3.3 Assessment of TDF adherence using TFV‐DP DBS concentrations

**Figure 1:**
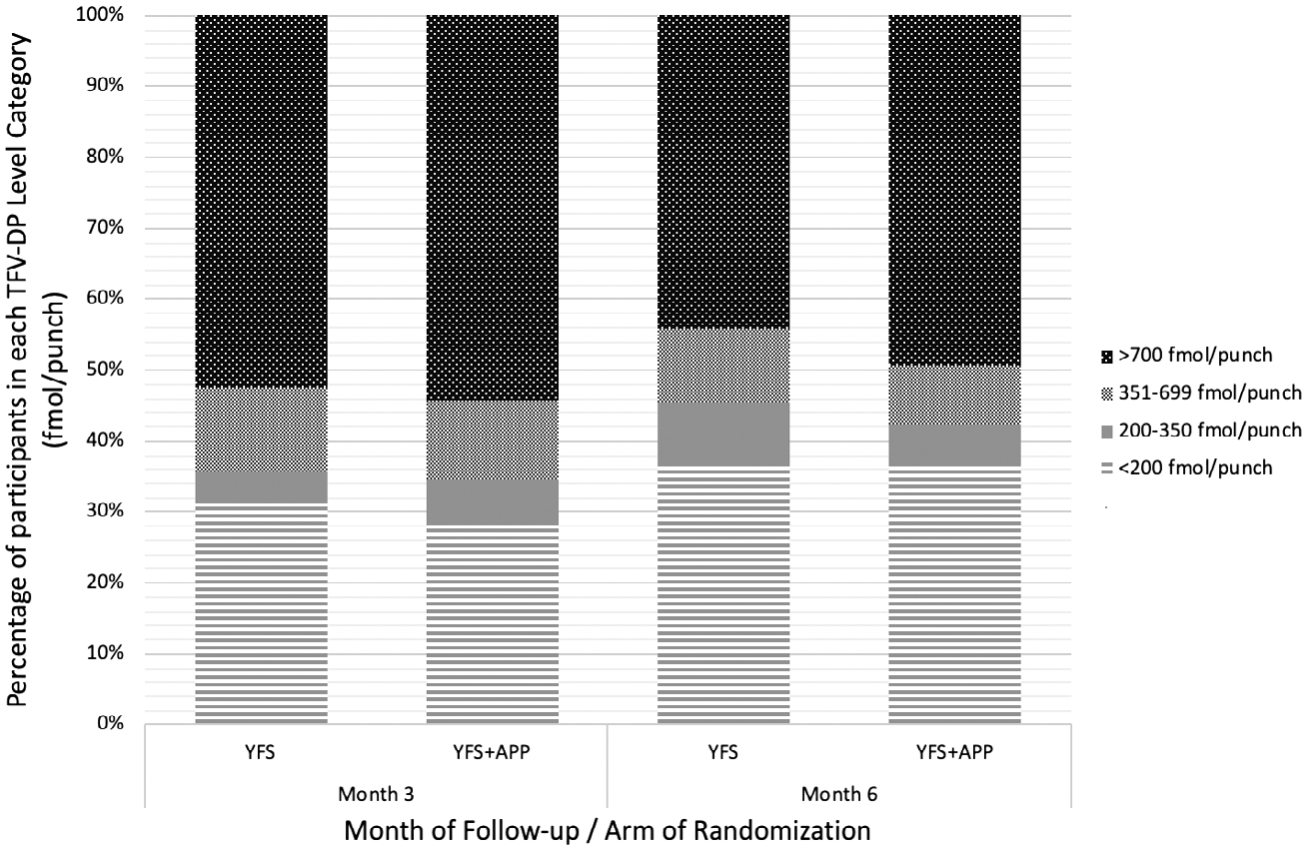
Adherence to once daily oral HIV pre‐exposure 3 prophylaxis at months 3 and 6 of follow‐up by randomization arm. YFS, Youth‐friendly services; YFS + APP, Youth‐friendly services plus mobile phone application use.

A total of 294 TFV‐DP samples were collected from 164 participants, 155 at month 3 and 139 at month 6. An additional seven timepoints (5 clients at month 3 and 2 clients at month 6) were added in the analysis as undetectable TFV‐DP levels, where blood samples were not taken due to self‐reporting of no PrEP use in the preceding month. A total of 301 risk periods were therefore analysed. PrEP adherence by TFV‐DP level was 52% (95% CI 45 to 60) at month 3 and 48% (95% CI 39 to 55) at month 6. PrEP adherence at month 3 was 51% in the YFS arm and 54% in the YFS + APP arm (*p* = 0.64) and at month 6 was 44% in the YFS arm and 49% in the YFS + APP arm (*p* = 0.54) (Figure 1).

### 3.4 Associated factors with PrEP adherence

Variables with significant association (*p* < 0.1) in univariable analysis to PrEP adherence were included in a further multivariable analysis, which included: gender identity, age, number of sex partners, self‐perceived risk for HIV infection and study arm. Education was not included due to its collinearity with age. Multivariable analysis showed only gender identity remained significantly associated with PrEP adherence at month 3, with YMSM being associated with greater odds of PrEP adherence compared to YTGW (OR 3.1, 95% CI 1.3 to 7.6, *p* < 0.01), but not at month 6 (OR = 2.4, 95% CI 1.0 to 6.2, *p* = 0.06) (Table 4).

**Table 4:** Associated factors with PrEP adherence^a^, 4A at month 3 of follow‐up 4B at month 6

	N	PrEP adherent^a^ N %		Unadjusted odds ratio (95% CI)	*p*‐value	Adjusted odds ratio (95% CI)	*p*‐value
Characteristics (Total N = 160)							
Month 3 follow‐up							
Total	160	84	52.5				
Gender identity							
MSM	120	74	61.7	4.83 (2.16, 10.79)	**0.003**	3.13 (1.30, 7.55)	0.01
TGW	40	10	25.0	1			
Age at enrolment(years)							
15 to 17	49	17	34.7	1			
18 to 19	111	67	60.4	2.87 (1.42, 5.77)	**0.003**	1.91 (0.87, 4.20)	0.11
Education							
≤Junior high school	70	30	42.9	1			
≥Senior high school	90	54	60.0	2.00 (1.06, 3.77)	**0.03**		
No. of sex partners in past month							
0	48	17	35.4	1			
1	70	40	57.1	2.43 (1.14, 5.20)	**0.02**	1.62 (0.69, 3.79)	0.26
≥2	42	27	64.3	3.28 (1.38, 7.80)	**0.007**	1.74 (0.66, 4.63)	0.26
Condom use in past month (N = 112)							
Consistent use	38	21	55.3	1			
Inconsistent use	74	46	62.2	1.33 (0.66, 2.94)	0.48		
Substance use in past three months^b^							
No	137	70	51.1	1			
Yes	23	14	60.9	1.49 (0.60, 3.67)	0.39		
Self‐perceived HIV acquisition risk (N = 157)							
Not at risk	96	42	43.8	1			
At risk	61	40	65.6	2.45 (1.26, 4.76)	**0.008**	1.74 (0.84, 3.63)	0.14
Laboratory diagnosed STI at baseline							
No	123	63	51.2	1			
Yes	37	21	56.8	1.25 (0.60, 2.62)	0.56		
Intervention							
YFS	79	40	50.6	1			
YFS + APP	81	44	54.3	1.16 (0.62, 2.16)	0.64	1.08 (0.54, 2.16)	0.82
Characteristics (total N = 141)							
Month 6 follow‐up							
Total	141	66	46.8				
Gender identity							
MSM	105	57	54.3	3.56 (1.53, 8.30)	**0.003**	2.44 (0.96, 6.20)	0.06
TGW	36	9	25.0	1			
Age at enrolment (years)							
15 to 17	42	12	28.6	1			
18 to 19	99	54	54.6	3.00 (1.38, 6.53)	**0.006**	1.89 (0.80, 4.50)	0.15
Education							
≤Junior high school	63	23	36.5	1			
≥Senior high school	78	43	55.1	2.14 (1.08, 4.22)	**0.03**		
No. of sex partners in past month							
0	41	16	39.0	1			
1	62	25	40.3	1.06 (0.47, 2.37)	0.90	0.80 (0.33, 1.91)	0.61
≥2	38	25	65.8	3.00 (1.20, 7.52)	**0.02**	1.94 (0.71, 5.30)	0.20
Condom use in past month (N = 100)							
Consistent use	33	17	51.5	1			
Inconsistent use	67	33	49.2	0.91 (0.40, 2.10)	0.83		
Substance use in past three months^b^							
No	118	57	48.3	1			
Yes	23	9	39.1	0.69 (0.28, 1.71)	0.42		
Self‐perceived HIV acquisition risk							
Not at risk	89	37	41.6	1			
At risk	52	29	55.8	1.77 (0.89, 3.54)	**0.10**	1.28 (0.60, 2.76)	0.52
Laboratory diagnosed STI at baseline							
No	107	51	47.7	1			
Yes	34	15	44.1	0.87 (0.40, 1.88)	0.72		
Intervention							
YFS	68	30	44.1	1			
YFS + APP	73	36	49.3	1.23 (0.64, 2.39)	0.54	1.18 (0.58, 2.42)	0.64

Bold value indicates *p* < 0.1. fmol/punch, femtomoles per 3 mm punch; MSM, men who have sex with men; TGW, transgender women; STI, sexually transmitted infection, laboratory confirmed and included syphilis, *Neisseria gonorrhoea* and *Chlamydia trachomatis*, the latter 2 from anal swab and urine samples; TFV‐DP DBS, tenofovir diphosphate dried blood spots; YFS, Youth‐friendly services; YFS + APP, Youth‐friendly services plus mobile phone application.

^a^ “PrEP adherent” defined as TFV‐DP DBS concentrations ≥700 fmol/punch [equivalent to ≥4 doses of TDF/week].

^b^ “substance use” defined as alcohol, sildenafil citrate and other recreational and illicit drug use including alcohol, amphetamines, methamphetamines, ketamine, poppers (volatile alkyl nitrates), marijuana and sildenafil citrate.

### 3.5 Overall HIV acquisition risk protection

**Figure 2:**
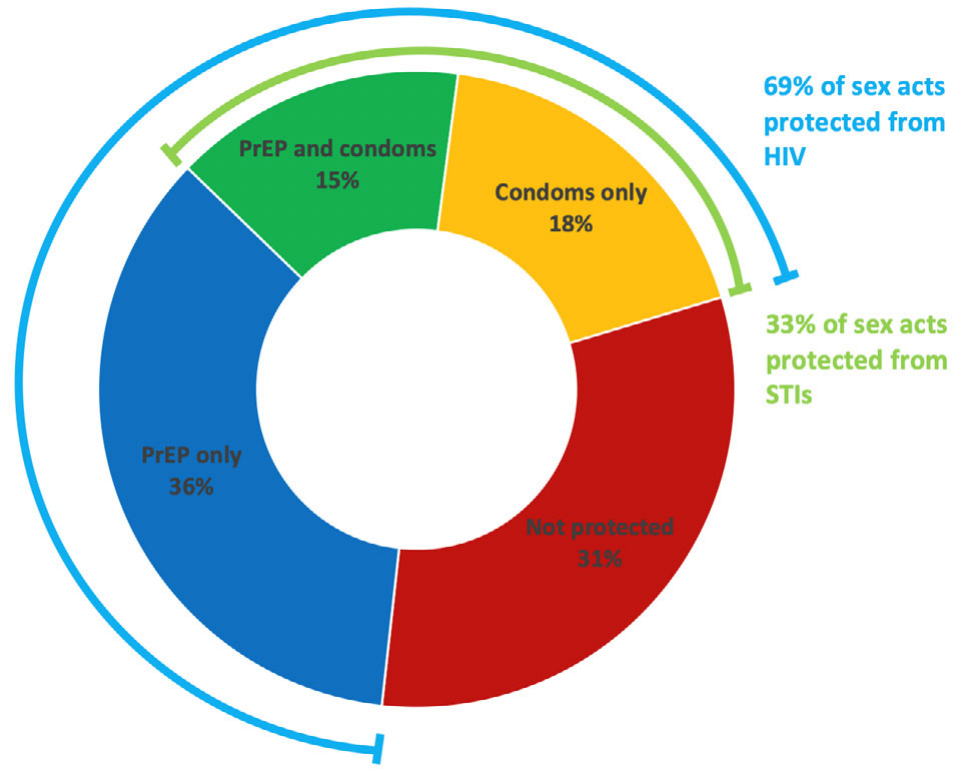
Mode of protection of risk periods against HIV and STIs seen in Thai adolescents. “PrEP protection” defined as ≥700 fmol/punch, “condom protection” defined as 100% condom use. PrEP, pre‐exposure prophylaxis; STIs, sexually transmitted infections.

A total of 296 risk period blocks for analysis were available (145 in YFS, 151 in YFS + APP, 5 of 301 available TFV levels had risk data missing so were excluded from this part of the analysis). In addition to the 51% of risk periods protected from HIV with PrEP, an additional 18% of risk periods were protected with self‐reported consistent condom use, achieving a presumed total of 69% protection against HIV (Figure 2). Of the 67 % of risk periods with inconsistent condom use reported, 54% of these were protected with PrEP.

## 4 DISCUSSION

To our knowledge, this is the first study in the Asia Pacific region that provides insights of youth‐friendly PrEP service implementation in YMSM and YTGW aged 15 to 19 years with an innovative approach using a mobile phone application to support adherence. This study demonstrated that youth‐friendly service integration to a PrEP programme enabled retention of three‐quarters of adolescent clients and HIV protective TFV‐DP levels of ≥700 fmol/punch among half of adolescent PrEP users during six months of follow‐up. However, the mobile application used did not provide additional PrEP adherence benefit in this randomized trial. For Thailand, lessons learned in this study are particularly valuable in supporting nationwide PrEP services for key populations including youth under its universal health coverage scheme as of October 2019, considerable progress from Thailand’s first fee‐based PrEP services in 2014 [29, 38, 39, 40, 41].

TFV‐DP DBS concentrations ≥700 fmol/punch, deemed to be protective from HIV for the preceding 30 days [42] in this study was found in 46% to 52% of participants at both three and six months, similar figures to those seen in US adolescent studies conducted between 2013 and 2014 (30% to 50%) at the same time points [21, 35]. An earlier adolescent study in 2010 that saw protective levels of only 20% by six months [43], a trend of improved adherence in newer studies also observed in adult studies, presumed to be due to higher general knowledge in PrEP efficacy and better informed and more motivated study participants in PrEP studies [44, 45]. The fact that all aforementioned studies including this study did not see any HIV seroconversions despite imperfect PrEP adherence may have been due to PrEP use being effectively used in an event‐driven fashion during risky events. Encouraging “prevention‐effective adherence,” the use of PrEP during periods of risk exposure to effectively protect against HIV acquisition is important because it acknowledges the dynamic pattern of HIV risk acquisition behaviours and the use of alternate HIV prevention strategies [46]. This may be a more appropriate goal to aim for in adolescents where the expectation of perfect adherence may become a barrier for those who would otherwise benefit from PrEP [44, 46]. We believe that in this study, a mix of few sex acts, use of alternate HIV protection methods such as condoms and reduced perceived risk of situations driven by incomplete neurocognitive development typical of the adolescence phase of growth contributed to the inconsistent PrEP use seen [44, 45, 47]. This emphasises the need to combine PrEP with condoms and other HIV prevention modalities as a package to protect adolescents against HIV. It is encouraging that approximately 50% of participants had “high” adherence based on TFV‐DP DBS concentrations of ≥700 fmol/punch and in combination with self‐reported consistent condom use increased the overall protection to 68%. This cohort showed that with service delivery of an HIV prevention package, an increase in proportion of condom use was seen, contrary to speculated concerns of PrEP reducing condom use [48]. However, it is acknowledged that condom use can only be measured by self‐report. We believe that recall bias was minimized by surveys at monthly intervals, although it should be kept in mind this may still have been offset with a degree of reporting bias due to social desirability bias.

The vulnerability to HIV infection of the population investigated was reflected by the high rates of self‐reported inconsistent condom use (66%) and laboratory diagnosed STIs at baseline of 23%, similar figures to those seen in the ATN studies 110 and 113 [21, 35]. This was in contrast to the majority of participants who felt at low or moderate risk of HIV infection. This suggests that HIV prevention services for adolescents may be better justified to offer services based on risk behaviours rather than self‐perceived risk. This cohort of adolescents had a relatively lower number of sex acts (2 to 3/month) and partners in the preceding month compared to much higher numbers (8 to 10/month) in the seminal ANRS Ipergay trial in adult MSM. This highlights the unique context of young people being at high risk of HIV acquisition for reasons such as lower rates of condom use and access to PrEP services, rather than number of sexual exposures [49]. For this reason, event‐driven PrEP may be more suited to adolescents, further studies are needed [50].

There are studies in adolescent HIV prevention research with preliminary data suggesting the feasibility and acceptability of apps in supporting PrEP adherence, however, trial results on efficacy are pending [51, 52, 53, 54]. The “Raincoat” app used in this trial did not show efficacy in PrEP adherence or service retention. The median time of app use was only three months out the total six months of the study, substantially lower than in an adult study where >60% continued to use a PrEP adherence app at by six months [55]. This may have been due to (1) lack of social networking features such as chat boards or a leader board, (2) lack of ongoing new features over the six‐month period contributing to boredom (3) availability on only one operating system (Android), meaning one‐third of participants had to borrow phones to use the app, which may have been inconvenient. Retention could further be addressed with more adaptive designs to service delivery, which include (1) *convenience* – more accessibility and flexibility of services, self‐testing for HIV and postal delivery of PrEP, (2) *adaptability to medication needs –* adolescent sexual activity changes over time and may be more suited to event‐driven PrEP, (3) *technological developments* to add interactive activity such as leader boards, and chat boards to increase engagement or sense of community.

There are some data to suggest risk perception influences risk behaviours in adult MSM [56, 57], and has similarly been described in a Thai YMSM study. We designed “Project Raincoat” based on the assumption that self‐risk assessment would lead to increased adherence. Findings from studies have been mixed with some evidence to suggest risk perception has some relation to reduced risk behaviours [58, 59], whereas in others despite risk perception, risk reduction was unaffected [60]. It could be argued that although there is evidence to suggest low perceived risk is associated with poor adherence in YMSM [61], this does not mean the opposite is necessarily true. It is important to point out that risk perception was part of a spectrum of other factors influencing risk taking behaviours not explicitly addressed by the intervention in this arm, such as addressing of barriers to condom use and alcohol use which may explain the outcomes observed in this study [62]. At the time of this study, there were no universally accepted validity tools available for the effectiveness of mobile applications prior to taking them to randomized trials. There is acknowledgement that multiple factors influence whether a mobile application is effective, including participant demographics, intervention factors (intensity, components), technology used (video, infographics, text) and behavioural change intervention used, only some of which were possible to investigate in this trial [63]. More understanding is also needed on whether and how point rewards influence YMSM behaviour. A qualitative component of this study is underway.

Retention in HIV prevention services was fairly high at 73% by six months, similar to figures seen in the ATN 110/113 studies, with no clear differences between YMSM and YTGW in contrast to previous experience in Thailand suggesting TGW have poorer retention rates in HIV prevention care [64]. This may have been related to comprehensive care including gender affirming hormone therapy [65]. High overall retention rates suggest that the approach to counselling and multidisciplinary care taken in this study were important in engaging and retaining adolescents in HIV prevention services as has been previously found [12].

The strength of this study was it provided youth‐friendly services that focused on adolescents between 15 and 19 years. This study was made possible through Thailand’s policies that allow adolescents aged 13 to 18 years to access to HIV and STI testing and treatment without parental consent and approval of parental consent exemption by an ethics committee. It was further supported by the Princess PrEP Program led by HRH Princess Somsawali, recently appointed UNAIDS Goodwill Ambassador for HIV Prevention in Asia and the Pacific (2019) that funded our PrEP supply in accordance with HIV acquisition risks regardless of age or gender identity [66]. These strengths of Thailand exemplify the advances possible in HIV prevention efforts through giving importance to the overcoming of legal and structural barriers. Finally, this study is continuing with qualitative data collection on the mobile phone application used, which will provide information to improve future designs.

Our study also had a number of limitations. The mobile application was only available on the Android platform. Additionally, this study was not specifically designed to focus on YTGW, who are increasingly recognised as a distinct group with unique needs and therefore it could be argued that a separate gender‐specific study with tailored interventions for YTGW is more appropriate [67]. The relatively short six‐month duration of follow‐up was also a limitation. Although this study looked at PrEP “execution,” further study is needed to address PrEP “persistence” in order to study ongoing HIV risk adolescents have over time [46]. We currently have an ongoing open‐label extension study following up participants to look at PrEP persistence. It must also be noted that the comprehensive YFS offered in this study is not routinely offered across Thailand and other middle‐income countries in the region, which may have negated any effects the mobile app had on PrEP adherence on the intervention arm of this study. Self‐reported measures regarding sex acts, sex partners or condom use may have been subject to reporting bias. Finally, this study may have been underpowered to detect differences in study arms due to a higher than expected dropout rate.

## 5 CONCLUSIONS

Using youth‐friendly service delivery, we were able to achieve retention in three‐quarters of our clients at six months and good adherence among half of adolescent PrEP users, with no seroconversions. However, the mobile phone application tested did not provide additional PrEP adherence benefit to comprehensive YFS in this randomized trial. Adolescent HIV risk behaviours are dynamic and require adaptive programmes that focus on “prevention‐effective adherence.” Further research is required on how improved adherence may be programmatically supported in adolescents both short and long‐term through further qualitative and PrEP persistence studies.

## COMPETING INTERESTS

The authors have no conflicts of interest to declare.

## AUTHORS’ CONTRIBUTIONS

WNS was responsible for writing the initial version of the manuscript. SK did the data analysis. TP supervised the overall manuscript writing process and content. NP and TC oversaw PrEP operations at the TRCARC. TRC led TFV‐DP laboratory analysis. PW coordinated this study and did the data collection. CS oversaw holistic care of study participants. SJ and DL oversaw PrEP operations at community‐based organisations. All authors have read and approved the final version of this manuscript.

## FUNDING

This study was supported by the CIPHER Grant ID 2017/472‐SON; The Princess PrEP Fund; FHI360; LINKAGES; The Chulalongkorn University Ratchadapisek Sompotch Fund, Faculty of Medicine (Grant number 61/037); The Chulalongkorn University Ratchadapisek Sompotch Fund (2019), Telehealth Cluster Grant; The Chulalongkorn University Ratchadapisek Sompotch Fund, Postdoctoral Fellowship Fund; The Center of Excellence in Transgender Health, Chulalongkorn University.
